# Reply to letter to the editor, “To screen or not to screen”

**DOI:** 10.1055/a-1931-3807

**Published:** 2022-10-17

**Authors:** Renata Nobre, Fauze Maluf-Filho

**Affiliations:** 1 Sao Paulo Cancer Institute (ICESP), Gastrointestinal Endoscopy Unit, Gastroenterology Department, Sao Paulo, Brazil

 We would like to take the opportunity to respond to the critical issues raised in the letter to the editor, “To screen or not to screen.” We would also like to thank Dr. van Tilburg and colleagues for their interest in our paper and for taking time to express their concerns.

In this letter to the editor, Dr. van Tilburg and colleagues questioned the cost and benefits of population-based screening for all patients with head and neck squamous cell carcinoma (HNSCC) and propose a risk-based patient selection.

Most patients with HNSCC are still diagnosed at an advanced stage (III and IV according the TNM system) 1 . Despite the advanced stage of their disease, most of them can benefit from modern multimodality treatment because great advances have been made in management of HNSCC in recent decades. The 5-year survival has improved when analyzed across all age groups and anatomical sites within the Surveillance, Epidemiology, and End Results (SEER) database 2 . As a result, it is reasonable to screen for a second primary tumor in this population.

We acknowledge the importance of individualizing the screening strategy according to performance status, life expectancy, and other factors associated with the HN primary tumor. The research setting of our study may limit the generalizability of the results. We also agree that risk stratification of patients with HNSCC would improve screening selection of high-risk patients.

Even though more than 4000 patients had been admitted to our hospital with HNSCC during the study period, about 32 % were excluded due to advanced disease with no curative intent treatment 3 . This fact reflects the late diagnosis and, consequently, the referral of more advanced cases to our quaternary academic center. Of the selected patients, even those with locally advanced HN disease or advanced age presented with low or moderate functional capacity, and we believe they could benefit from screening.

As mentioned by Dr. van Tilburg and colleagues, according to Max Wilson 4 , requirements for screening involve adherence to treatment, facilities for diagnosis and treatment, recognizable latent or early symptoms, suitable test or examination, natural history adequately understood, agreed upon policy for treatment, and costs related to other medical care expenditure and continuing the process. We believe that most of these requisites are fulfilled by the screening program for ESCC in patients with HNSCC, because endoscopy can detect early-stage cancer and, therefore, significantly improve the survival rate, as shown by our results. We agree that, despite this evidence, the effectiveness of a screening program should consider costs. However, the primary objective of our research was to compare survival rates, and we believe that further studies that examine the cost-effectiveness of population risk-based screening and surveillance are needed.

In summary, we believe that our results support the annual screening program for ESCC in patients with HNSCC and that targeting higher-risk individuals can improve clinical outcomes and reduce costs.
